# Closely related Lak megaphages replicate in the microbiomes of diverse animals

**DOI:** 10.1016/j.isci.2021.102875

**Published:** 2021-07-16

**Authors:** Marco A. Crisci, Lin-Xing Chen, Audra E. Devoto, Adair L. Borges, Nicola Bordin, Rohan Sachdeva, Adrian Tett, Allison M. Sharrar, Nicola Segata, Francesco Debenedetti, Mick Bailey, Rachel Burt, Rhiannon M. Wood, Lewis J. Rowden, Paula M. Corsini, Steven van Winden, Mark A. Holmes, Shufei Lei, Jillian F. Banfield, Joanne M. Santini

**Affiliations:** 1 Institute of Structural and Molecular Biology, Division of Biosciences, University College London, London, UK; 2 Department of Earth and Planetary Science, University of California Berkeley, Berkeley, CA, USA; 3 Innovative Genomics Institute, University of California Berkeley, Berkeley, CA, USA; 4 Department CIBIO, University of Trento, Trento, Italy; 5 Bristol Veterinary School, University of Bristol, Langford, Bristol, UK; 6 Department of Veterinary Medicine, University of Cambridge, Cambridge, UK; 7 Zoological Society of London, London, UK; 8 Quadram Institute Bioscience, Norwich Research Park, Norwich, UK; 9 Royal Veterinary College, London, UK; 10 The University of Melbourne, Melbourne, VIC, Australia

**Keywords:** Virology, Microbiome, Omics

## Abstract

Lak phages with alternatively coded ∼540 kbp genomes were recently reported to replicate in *Prevotella* in microbiomes of humans that consume a non-Western diet, baboons, and pigs. Here, we explore Lak phage diversity and broader distribution using diagnostic polymerase chain reaction and genome-resolved metagenomics. Lak phages were detected in 13 animal types, including reptiles, and are particularly prevalent in pigs. Tracking Lak through the pig gastrointestinal tract revealed significant enrichment in the hindgut compared to the foregut. We reconstructed 34 new Lak genomes, including six curated complete genomes, all of which are alternatively coded. An anomalously large (∼660 kbp) complete genome reconstructed for the most deeply branched Lak from a horse microbiome is also alternatively coded. From the Lak genomes, we identified proteins associated with specific animal species; notably, most have no functional predictions. The presence of closely related Lak phages in diverse animals indicates facile distribution coupled to host-specific adaptation.

## Highlights

• PCR and genome-resolved metagenomics revealed Lak phages in 13 animal species
• Lak is abundant in the pig hindgut, with potential relevance to growth performance
• Thirty-four new Lak phage genomes present expanded size range and phylogeny
• Unique Lak protein families from specific microbiomes suggest animal host adaptation

## Graphical abstract

**Figure:**
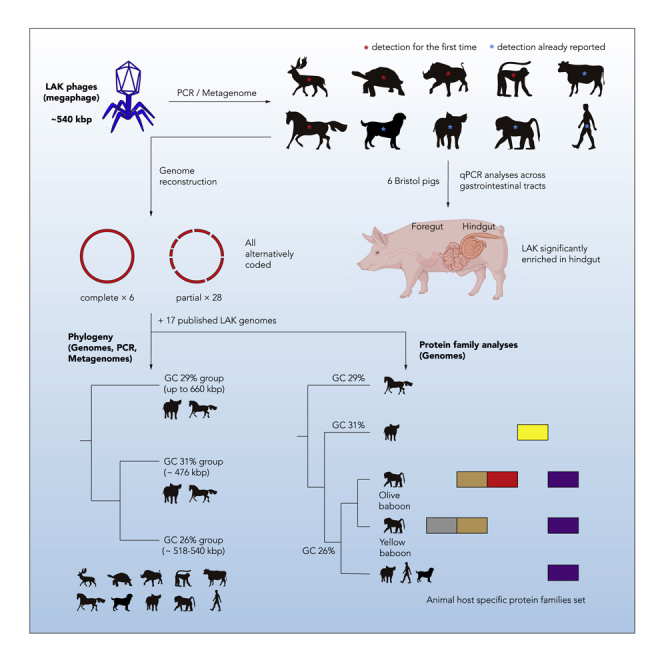

## Introduction

*Prevotella* and *Bacteroides* (phylum Bacteroidetes) occupy similar ecological niches and compete for resources in gut microbiomes (Gorvitovskaia et al., 2016; Ley, 2016). *Prevotella-* and *Bacteroides*-dominated enterotypes are linked to non-Western and Western diets, respectively (De Filippo et al., 2010; Ou et al., 2013; Tyakht et al., 2014; Wu et al., 2011). Diets low in fat and protein but high in fiber promote *Prevotella* growth, whereas diets high in animal fat, protein, and starch promote *Bacteroides* growth (Ley, 2016; Wu et al., 2011). *Prevotella* can metabolize fiber and produce volatile fatty acids that are crucial to gut health more effectively than *Bacteroides* (Chen et al., 2017). *Prevotella* is also widespread in pig gut microbiomes and generally associated with improved growth performance, an observation of interest because pigs are important production animals and model for the human gut (Mach et al., 2015; Ramayo-Caldas et al., 2016; Xiao et al., 2016).

Lak megaphages that replicate in *Prevotella* were recently discovered in human and baboon gut microbiomes using genome-resolved metagenomics (Devoto et al., 2019). To date, these phages are among the largest identified in gut microbiomes (>540 kbp genomes in length) and encode a tail sheath protein which (along with large terminase phylogeny) suggests a myovirus morphotype. Lak phage sequences were also detected in Danish pig metagenomes abundant in *Prevotella* and in cow rumens at low abundance (Devoto et al., 2019). Unlike smaller *Prevotella* phages that typically adopt a temperate lifestyle (Benler et al., 2021; Gregg et al., 1994), Lak genomes do not contain identifiable integrases and no prophages have been detected in bacterial chromosomes. Thus, it is likely that Lak phages are virulent. Lysis by Lak phages could alter the composition and abundance of *Prevotella* in the animal/human host*,* affecting microbial community structure and nutrient availability.

A notable feature of Lak is the use of an alternative genetic code, where the “TAG” stop codon is repurposed to encode glutamine (Q) (Devoto et al., 2019). Lak genomes encode a suppressor tRNA with a CTA anticodon needed to repurpose TAG. Moreover, the presence of release factor 2 (RF2) terminates protein translation through recognition of TGA and TAA stop codons but not TAG. The reason for Lak phage codon reassignment is unknown, but it may disrupt the translation of bacterial genes (Ivanova et al., 2014).

In this study, we screened digesta/fecal and mucosal samples from a wide variety of animals that consume dietary fiber to determine the distribution, genomic characteristics, and relatedness of the Lak phages detected. We also quantified the abundance of Lak phage and *Prevotella* across the swine gastrointestinal tract (GIT) and vagina by quantitative polymerase chain reaction (qPCR) and confirmed detection in proximal spiral samples via metagenomics. From pig and horse metagenomes, we manually curated six new Lak genomes to completion, substantially expanding the genome size range. From 34 new partial and complete Lak genomes, bacterial hosts and evolutionary relationships were predicted, and the extent of alternative codon usage among Lak phages evaluated. Protein family analyses were performed on all new and published Lak genomes (Devoto et al., 2019; Edgar et al., 2020) to identify animal-specific protein clusters that may be important for the adaptation of Lak phages to their microbiome environments.

## Results

### Lak phages detected in various animal microbiome samples by PCR

PCR primer sets targeting genes for the major capsid protein (MCP), tail sheath monomer (TSM), and portal vertex protein (PVP) detected Lak in 112/194 samples from many animal gut microbiomes (Table 1, Tables S1 and S2). Lak was detected in 80% of pigs (*n* = 28) but was undetectable in gestating sows (*n* = 4) and a post-farrow sow (*n* = 1) with piglets (*n* = 2). Jejunal and ileal (foregut); and proximal spiral, distal spiral, caecal and rectal (hindgut) lumen; and mucosal samples from six finisher pigs tested positive and samples were subjected to qPCR quantification. Three of five vaginal samples tested positive from pigs where Lak was detected in the rectum but not in the lungs, although *Prevotella* 16s rRNA genes were detected at all body sites by PCR. Lak signature genes were also detected in microbiomes of horses, a cow, giant tortoises, a fallow deer, and white-naped mangabeys. A subset of PCR products from each animal cohort was sequenced, confirming the presence of Lak (Table 1, Table S1).

**Table 1:** Lak phages detected in various animal microbiome samples by PCR

Animal	Sample type	Details	PCR positive
Cow (*Bos taurus*), Holstein	Rumen Fluid	1 Individual, female, ~10 years	1/3
Warthog (*Phacophoerus africanus*)	Feces	Group of 2, pooled	1/3
White-naped mangabey (*Cercocebus lunulatus*)	Feces	Group of 7, pooled	1/3
Galapagos giant tortoise (*Chelanoidis nigra*)	Feces	Adult group of 3 and juvenile group of 3, pooled separately	2/2
Fallow deer (*Dama dama*)	Feces	1 Individual, wild	1/1
(Epsom) horse (*Equus ferus caballus*), Racing thoroughbred	Feces	6 individuals over 3 days, various sexes and ages	12/18
(Hersham) horse (*Equus ferus caballus*), Welsh cob	Feces	1 Individual, female, ~20 years	1/1
(Royal Veterinary College) horse (*Equus ferus caballus*), Blood donor	Feces	3 Individuals, various sexes and ages	3/3
(Cambridge) Pig (*Sus scrofus*), large White/Landrance/Hampshire	Feces	19 Individuals, various sexes and ages	12/19
(Bristol) Pig (*Sus scrofus*), Welsh and Welsh/Petrain	Lumen digesta and mucosal scrapings^a^	6 Individuals, finisher pigs, various sexes	70/70
(Bristol) Pig (*Sus scrofus*), Welsh/Petrain	Vaginal and rectal samples^b^	5 Individuals, finisher pigs, female	8/10
(Royal Veterinary College) Pig (*Sus scrofus*), Large White/Unknown cross	Feces	Various groups, pooled	5/5

A subset of PCR products from each cohort were sequenced to confirm the presence of Lak. See details in Tables S1 and S2.

a Post-mortem pig samples from: foregut (jejunum and ileum) and hindgut (cecum, proximal spiral, distal spiral and rectum).

b Lak was detected in all rectums (n = 5) and 3/5 vaginal mucosa, but not in lungs of the same animals.

### Lak phage and *Prevotella* abundance differs across the pig GIT and vagina

**Figure 1:**
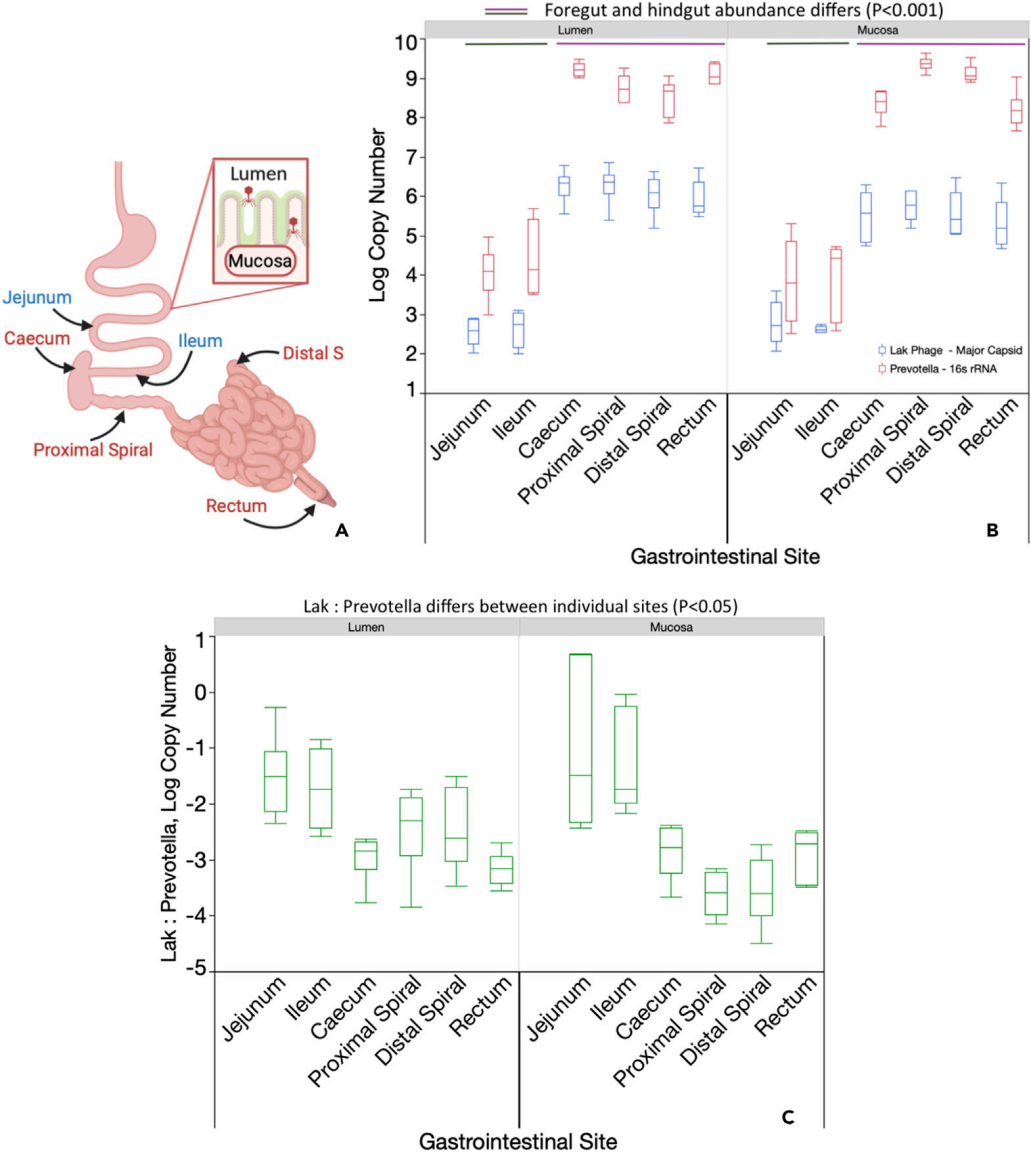
Lak phage and *Prevotella* abundance differs across the pig gastrointestinal tract (A) Schematic of pig GIT with labels indicating the sites sampled: Blue labels = foregut, Red labels = hindgut (main sites of microbial fiber fermentation). For both (B) and (C), Lak phage major capsid and *Prevotella* 16S rRNA gene copy numbers determined by absolute quantification qPCR, with 10 ng pooled DNA from each GIT site from 6 finisher pigs; for all sites except ileal lumens, where digesta was only present in 4/6 pigs. Top and bottom whiskers = minimum and maximum values. Box width = Interquartile range (IQR). Significant differences in Lak, *Prevotella* and Lak: *Prevotella* ratio means were determined by Tukey's HSD test. (B) Difference in Lak phage abundance across pig lumen and mucosal sites coincides with *Prevotella* 16S rRNA gene abundance. Solid green and pink lines represent differences in abundance deemed statistically significant (*P* < 0.001). Standard errors ranged from 0.17–0.21 (Lak), and 0.28–0.34 (*Prevotella*). (C) Difference in ratios of Lak phage to *Prevotella* 16S rRNA gene copies (*P* < 0.05). Standard errors ranged from 0.28–0.35.

The abundance of Lak and *Prevotella* was quantified in triplicate across the GIT of the Bristol finisher pigs (Table 1, Figure 1). Lak abundance correlated with that of *Prevotella* across the entire GIT, although there were fewer Lak phage MCP than *Prevotella* 16s rRNA gene copies at all sites (Figure 1B). Together, GIT site and sample type (mucosa or lumen) had a significant effect on Lak (*F*_5,54_ = 2.99, *P* = 0.019) and *Prevotella* (*F*_5,54_ = 4.24, *P* = 0.003) abundance. Foregut Lak and *Prevotella* abundance (jejunum and ileum) was significantly lower than that in hindgut sites (cecum, proximal spiral, distal spiral) in both the lumen and mucosa (*P* < 0.01, Tukey's HSD; Figure 1B). However, there was no statistically significant difference in both Lak and *Prevotella* abundance between the lumen and mucosa at each GIT site (*P* > 0.05, Tukey's HSD; Figure 1B). The ratio of Lak: *Prevotella* log copy numbers did not differ between mucosa and lumen at each site, but generally, was significantly higher in the foregut mucosa (jejunum = 0.076; Ileum = 0.041) than in hindgut lumen and mucosal sites (range = 0.0002–0.003; *P* < 0.05, Tukey's HSD; Figure 1C). The ratio of Lak: *Prevotella* was also higher in foregut vs. hindgut lumens, except in the cecum. Within the all-female pig group, Lak (*t*_1_ = 8.61, *P* = 0.0001) and *Prevotella* (*t*_1_ = 4.60*, P* = 0.0002) were more abundant in the rectum than in the vagina, although the Lak: *Prevotella* ratios were similar (Table S3).

### Newly reconstructed Lak phage genomes reveal expanded size range and genomic diversity

Eight new Lak phage genomes were reconstructed from new metagenomic data sets for a subset of samples identified to contain Lak, and 28 were reconstructed from published metagenomic datasets. Of these, 8 came from pig fecal samples (including one from a published data set of Danish pig, Pig_ID_1901_F52), 18 from human fecal samples, 7 genomes from baboon fecal samples, and one from a horse fecal sample (Table 2). Six of these 34 draft genomes were manually curated to completion. Two genomes are ∼476 kbp in length (476,085 and 476,118 bp, representative of a set of 4 genomes with GC contents of ∼31% from pig gut microbiomes), one genome is 517,629 bp in length (GC content ∼26% from a pig, detectable by read mapping at ≥ 5X coverage in 38.4% of previously reported pig metagenomes (Munk et al., 2018)), and one 659,950 bp genome (GC content ∼29% from a horse fecal sample; Table 2, Table S1). These findings substantially expand the known range of genome sizes and genomic diversity for Lak phages (Figure S1). The ∼476-kbp (GC31), ∼518-kbp (GC26) and ∼540-kbp (GC26) genomes are syntenic, and small blocks of sequence account for the differences in genome lengths. However, the 660-kbp phage genome is too divergent at the nucleotide level to align with those of the other clades, and its classification as Lak is based on the phylogeny of Lak proteins (see section “Lak phage genomes exhibit conserved, lineage-specific and animal-specific protein families”).

**Table 2:** Newly reconstructed Lak phage genomes reveal expanded size range and genomic diversity

Animal source	Microbiome type	No. of genomes/complete genomes	No. of scaffolds/genome length	GC (%)
Human	International Human Microbiomes – fecal samples from China	3/0	2–18/445–540 kbp	~26
	International Human Microbiomes – fecal samples from Denmark	2/0	2–17/478–537 kbp	~26
	International Human Microbiomes – fecal samples from Spain	9/0	4–20/408–528 kbp	~26
	Human Gut Microbiome – fecal samples from China, Israel, Italy, Liberia	4/0	1–8/499–544 kbp	~26
Baboon	Yellow Baboon fecal samples	3/0	1–6/544–546 kbp	~26
	Olive Baboon fecal samples	4/0	3–30/537–545 kbp	~26
Pig	Fecal	4/3	1–26/517–541 kbp	~26
	Fecal or *Prevotella* infection enrichment	4/2	1–9/463–479 kbp	~31
Horse	Individual fecal sample	1/1	1/660 kbp	~29

All new Lak genomes reconstructed in this study are listed, which were included for protein family analyses, along with the 15 published Lak genomes (Devoto et al., 2019) and all the 181 circular huge phage genomes reported recently (Al-Shayeb et al., 2020). See details in Table S1.

### 660-kbp Lak phage detected in additional horses and shows day-to-day variation

Specific PCR and qPCR assays targeting the MCP gene were designed to investigate the 660 kbp Lak discovered in horse B. The Lak phage could not be detected in 11 fecal DNA samples from various zoo ungulates or any of the other Epsom racehorses (Table S1). However, the 660-kbp Lak MCP gene was detected in an additional horse from a separate stable (G) and three RVC horses (H, I and J). These 4 additional horses did not test positive using the general Lak PCR primer sets (Table S1). Lak phage abundance in horse B declined from day 1 to day 3 (Table S4). Abundance of the *Prevotella* genus remained constant and did not correlate with the decline in Lak phage abundance (*r*_1_ = 0.048, *P* =0.970). Abundance of Lak phage and *Prevotella* in horse G feces at the single time point was comparable to results from horse B on day 3.

### Lak phages from diverse animals are phylogenetically related

**Figure 2:**
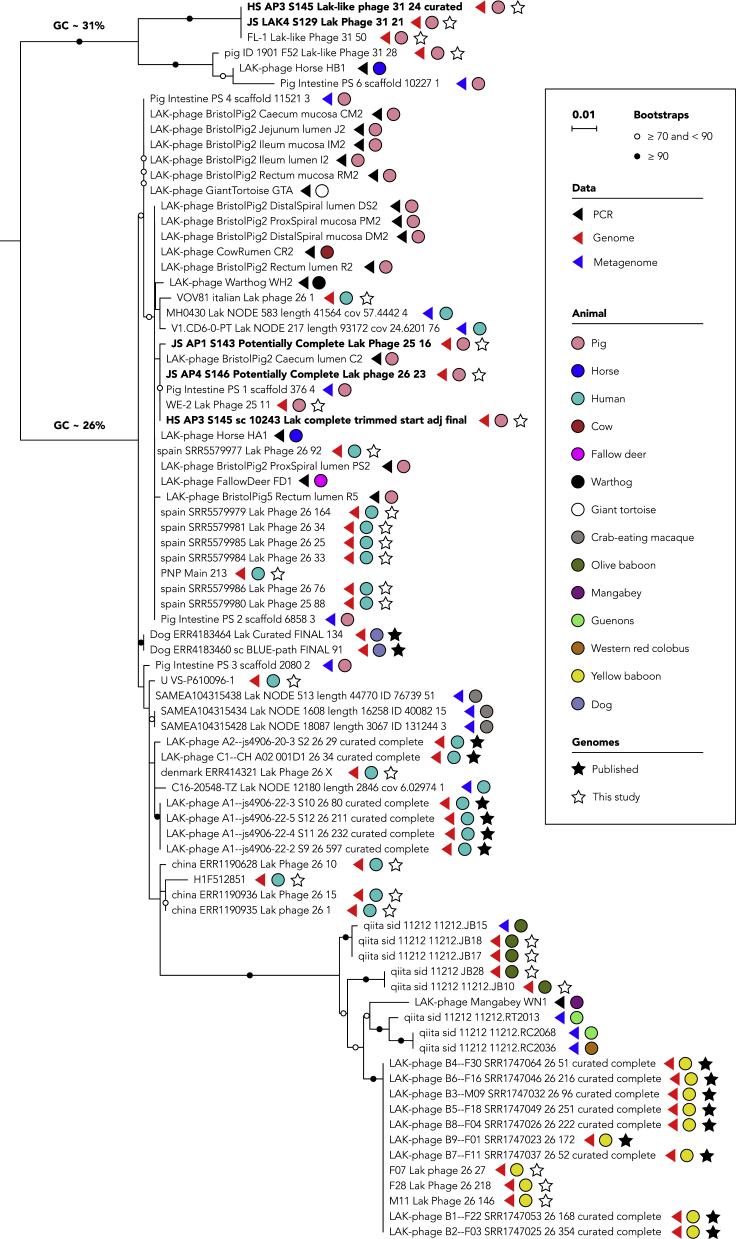
Lak phages from diverse animals are phylogenetically related Phylogeny was based on sequences from PCR, genomes, and metagenomes. The nucleotide sequences encoding the major capsid protein (MCP) were aligned and trimmed so that all lengths corresponded with that of the PCR-derived sequences. The capsid of the ~660-kbp phage is very divergent from others, thus was excluded from the tree to enable resolution of the other sequences. The tree was rooted between the GC31 group and GC26 group, according to the full phylogeny of all Lak (including the ~660-kbp one) and some other published huge phages (Figure S6). Three partial Lak phage genomes do not contain the MCP sequences thus were excluded. The names of the complete Lak genomes reported in this study are in bold. Bristol pig sequences obtained from the vaginal mucosa were identical to those found in the digestive tract. Corresponding trees for portal vertex and tail sheath monomer genes are shown in Figures S2 and S3.

To investigate the relatedness of Lak phages, phylogenetic trees were constructed based on the PCR-amplified, genome-derived and metagenome assembled (and not binned) nucleotide sequences of Lak MCP (Figure 2), TSM, and PVP genes (Figures S2 and S3). With all conserved genes, we found that the Lak phages from olive baboon, mangabey, guenons, western red colobus, and yellow baboon were more phylogenetically related. The Lak phages from horses, warthog, giant tortoise, cow, fallow deer, and most pig microbiomes were generally clustered together on the trees. Moreover, the Lak phages detected in crab-eating macaques were closely related to some from human microbiomes (Figure 2).

### Newly reported Lak phages are predicted to replicate in *Prevotella*

We analyzed all of the detected CRISPR-Cas systems from the scaffolds of the corresponding samples. For a given scaffold with a CRISPR-Cas system identified, all spacers from the scaffold and also the reads that mapped to it were extracted to search for their targets (≥90% identity; STAR Methods). We found that the pig-derived WE-2_Lak_Phage_25_11 was targeted by three spacers (total count = 11) from WE-2_scaffold_6241 (total count = 89, unique spacers count = 38; Figure S4). The genome of denmark_ERR1305877_Lak_Phage_26_8 was targeted by two unique spacers (total count = 3), which were respectively from two CRISPR-Cas systems on two scaffolds. None of the scaffolds were binned to a genome, but most of the genes on them had the highest similarity to *Prevotella* genes. The indication that these newly reported Lak phages had infected *Prevotella* is consistent with the previous finding (Devoto et al., 2019) that Lak are targeted by CRISPR spacer matches from *Prevotella* in human gut microbiomes. This putative host is currently classified as CAG 386 which is in the species-level “Clade B” of the *Prevotella copri* complex (Tett et al., 2019). We also detected no integrases by functional annotation, corroborating previous findings that Lak phages do not integrate into host genomes (Devoto et al., 2019).

### Alternative coding is a persistent feature of the expanded Lak phage clade

Although we anticipate that Lak phage genomes use genetic code 15 (only TGA and TAA are stop codons), we first predicted the Lak phage genes using code 11 (in which TAG, TGA and TAA are read as stop codons) to check the expanded dataset for evidence of alternative coding. For all Lak, the coding density was consistently low when genes were predicted using code 11, indicating a stop codon reassignment. Reprediction without the use of the TAG stop codon (as in code 15) resulted in full-length open reading frames. However, even after reprediction using code 15, some regions still had low coding density (many regions >1 kbp and some >2 kbp with no predicted open reading frames), extending our prior findings of low coding densities in other Lak phages (Devoto et al., 2019).

**Figure 3:**
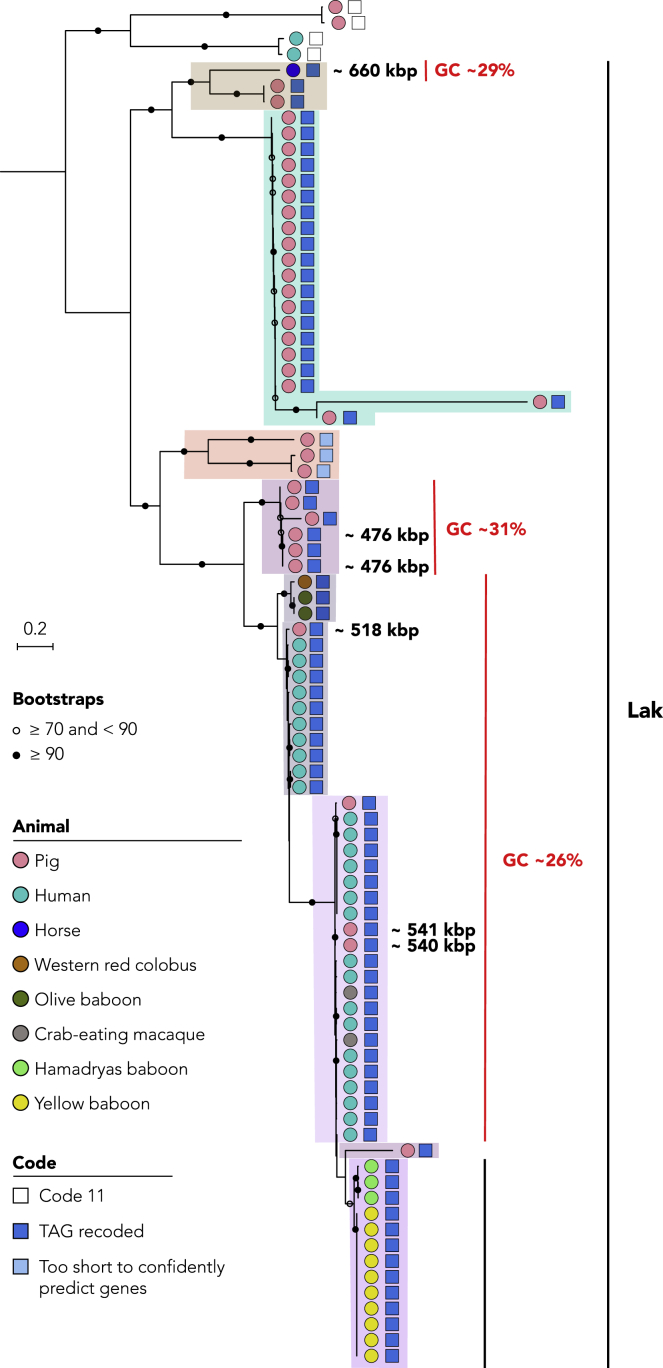
Alternative coding is a persistent feature of the expanded Lak phage clade The maximum likelihood phylogenetic tree (iqtree (v1.6.12) using the "LG+G4" model (-bb = 1000)) was constructed using sequences for the large terminase protein sequence (see STAR Methods). The genome sizes shown are based on those of complete Lak phages in each clade. Nodes with ≥90% bootstrap support values are indicated by filled black circles and nodes with 70–90% support by open circles. Recoding of the TAG stop codon was detected through the Lak lineages but not in phages represented by the deepest branch.

To determine the phylogenetic span of alternative coding in Lak phages, we searched the metagenome data sets for the Lak large terminase proteins (whether or not they were on genome fragments assigned to bins) (Figure 3). The terminase proteins were highly fragmented when TAG was read as a stop codon. Coding was uncertain for one group of Lak phages, represented by very short genome fragments (pale blue boxes; Figure 3). However, results generally indicate that alternative coding persisted from the common Lak phage ancestor. The deepest branches in Figure 3 represent phages that show no evidence of recoding.

**Figure 4:**
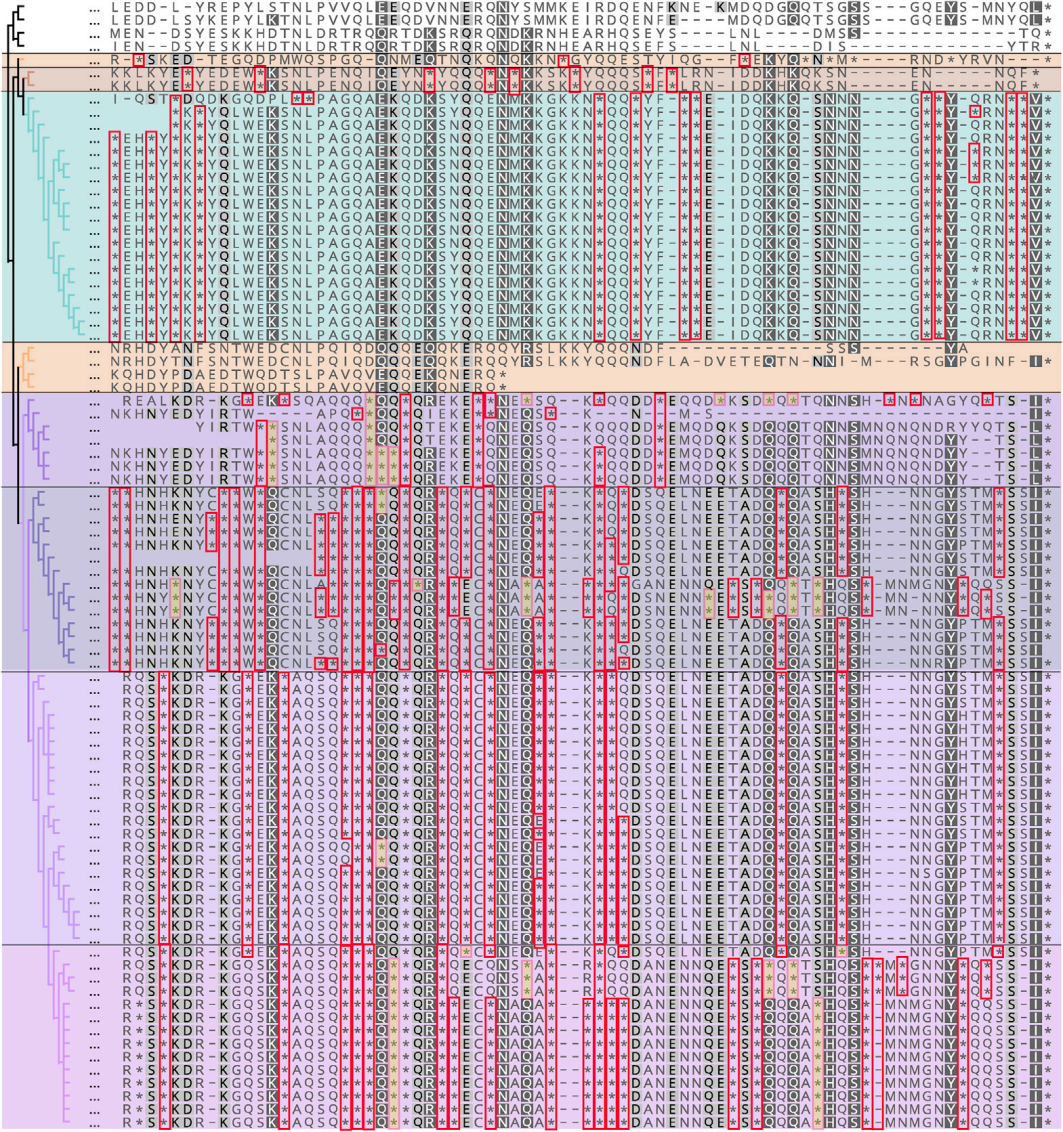
Compressed version of the large terminase protein sequence alignment in which all positions except those with in-frame TAG codons (represented by ∗) have been deleted Background shading indicates different Lak phage lineages, as shown in Figure 3). Colors superimposed on ∗ indicate positions in which there is within-clade consensus as to the identity of the aligned amino acid. In the Lak clades with ~26% GC (bottom three groups), Q is the aligned amino acid in 77%, 75% and 85% of cases. There is insufficient information in other groups to predict how TAG is translated.

Previous analyses of Lak (Devoto et al., 2019) and other alternatively coded phages (Ivanova et al., 2014) suggested that TAG is recoded to glutamine (Gln, Q). However, prior studies did not investigate variation in TAG codon use patterns within genes or consider the possibility of alternative translations. Thus, we aligned terminase sequences where TAG is represented as ∗ to identify the aligned amino acids for each clade (Figure 3). Based on cases where one specific amino acid in at least two different sequences was aligned with one or more ∗ (Figure 4), we deduced that throughout much of the Lak clade, TAG is likely translated as Q. These in-frame TAGs were probably introduced by synonymous substitution, i.e., CAG (Q) to TAG. In some cases, ∗ aligned with E (glutamic acid), which is chemically similar to Q (Figure 4). Plausibly, this occurred by mutation of GAG to TAG. Within the four Lak lineages (shaded in Figures 3 and 4), positions with only ∗ within and across clades may be mutations that introduced TAG after the rise of alternative coding in the ancestral group. Due to low information content, the alignment could not resolve the translation in three clades (green, orange, and brown shading in Figures 3 and 4).

### Suppressor tRNAs facilitate alternative coding in Lak phages

Stop-codon reassignment can be facilitated by the acquisition of a suppressor tRNA to decode the reassigned stop codon as an amino acid. To define the tRNA repertoire of the expanded Lak clade, we searched the high-quality Lak genomes for tRNAs with tRNAscan-SE (Chan and Lowe, 2019). Lak phages encode 24 to 56 tRNAs (Table S5), and the majority of them (38/51) encode 1–2 copies of a suppressor tRNA predicted to decode the TAG stop codon. Notably, these phages also universally (51/51) encode a suppressor tRNA predicted to bind the TAA stop codon. However, we find no other evidence to suggest the TAA stop codon is also recoded in Lak phages.

### Lak phage genomes exhibit conserved, lineage-specific, and animal-specific protein families

**Figure 5:**
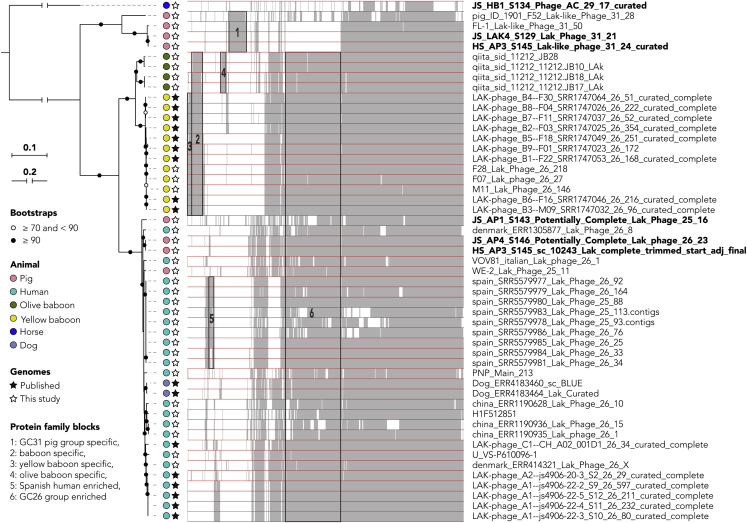
Lak phage genomes exhibit conserved, lineage-specific and animal-specific protein families Phylogenomic analyses of the 51 Lak phage genomes were performed. The phylogenetic tree (left) was built based on concatenated sequences of 49 single copy protein families detected in all Lak genomes and re-rooted using the sequence of the ~660-kbp horse-associated Lak phage. The protein family content heatmap (right), aligned with the phylogenetic tree, shows the presence/absence of protein families that could be detected in at least 4 genomes. The names of the 6 complete Lak genomes reported in this study are in bold. A total of 6 blocks of protein families with group-specific or animal-specific distribution patterns are highlighted in boxes and numbered.

We clustered predicted protein sequences into protein families and examined the distribution of families across 51 high quality Lak genomes to investigate whether, and to what extent, Lak phages have a conserved core gene set and if some genes are specific to Lak phages found in gut microbiomes of certain types of animals (Figure 5). The protein family analyses were performed for the 34 newly reconstructed (Table 2) and the 17 published Lak genomes (Devoto et al., 2019; Edgar et al., 2020) and the 181 circularized huge phage genomes reported recently (Al-Shayeb et al., 2020). Clustering analyses grouped the ∼660-kbp phage with other Lak phages, although it has a very divergent protein family profile (Figure S5), which is consistent with the phylogeny based on the protein sequences of MCP (Figure S6). A total of 221 protein families were detected in at least 49 out of the 51 Lak genomes (referred to as “Lak_core”; Table S6). Among “Lak_core'' protein families, 108 were only present in Lak genomes (i.e., Lak-specific). Only 3 Lak-specific protein families could be annotated (i.e., magnesium transporter [K03284], protein transport protein SEC20 [K08497], and a pyruvyltransferase-like protein [K13665]). Interestingly, the pyruvyltransferase-like protein contains two domains (Glyco_tranf_2_4 and PS_pyruv_trans), both of which have the highest similarity to those from *Prevotella* species. A total of 113 “Lak_core” protein families are also present in non-Lak phage genomes. They are generally phage structural proteins, including large terminase, prohead core protein, baseplate wedge subunits, neck protein and tail tube protein etc., and those for replication, recombination and repair including HNH nucleases, DUTP diphosphatase, DNA polymerase, DNA primase, RecA/RadA recombinase, and ribonucleoside-triphosphate reductase (Table S6).

We detected some protein families in Lak genomes that are only found in specific animal hosts (Figure 5). For example, 18 protein families were only detected in baboon Lak genomes, three only in olive baboon Lak genomes and 6 only in yellow baboon Lak genomes. Also, we found 37 protein families in all four genomes of the pig-associated GC31 group (including UK and Danish pigs) but in no other Lak genomes. We speculate that these animal host-specific protein families could be important during infection of their animal-specific *Prevotella* species and/or adaptation to the animal host. However, the inability to assign functions to these proteins at present hinders our understanding of their biological roles (Table S6).

## Discussion

### Lak phages are prevalent across diverse human and animal microbiomes

Here, we show that Lak phages are present in microbiomes of humans from China, Denmark, Italy, Spain, Israel, and Liberia, various pig breeds, non-human primates (white-naped mangabey, yellow and olive baboons, macaques, guenons and colobus), horses, warthogs, fallow deer, a cow rumen, and Galapagos giant tortoises (first reported from a reptile), which likely had similar microbiome composition to hindgut-fermenting mammals (*Bacteroidetes-* and *Firmicutes-*dominated) (Sandri et al., 2020; Yuan et al., 2015). Additionally, Lak phage genomes were recently resolved from two Labrador retriever metagenomes from a different study (Allaway et al., 2020; Devoto et al., 2019; Edgar et al., 2020), but Lak was undetectable in other dogs by PCR (Table S1). Lak was mainly detected in monogastric (single-chambered stomach) hindgut fermenters but also in some ruminants (cow and deer). The genome of one Lak from a horse microbiome is notable because it is now the largest Lak genome (659,950 bp). We are confident regarding this expanded genome size range because key genomes (including the largest) were manually curated to completion. Overall, our findings demonstrate that closely related Lak phages are widely prevalent in microbiomes of humans and animals.

Generally, phylogenies group together Lak phages that inhabit humans and some pigs, separating them from phages from other pigs (i.e., GC31 group), and from phages in non-human primates (and likely other animals based on PCR sequences) (Figure 2, Figures S2 and S3). The two dog Lak phages are related to human Lak. In addition to the 660-kb Lak genome (from HB2), nucleotide sequences suggest that other genotypic variants recovered from horses (from HB1 and HB2, PCR-derived) are similar to Lak from humans and some pigs. Furthermore, two GC content distinct Lak phages were detected within one pig. Multiple Lak variants may therefore occupy the same animal host. The newly constructed Lak phage genomes (including the most divergent from Horse B) are genetically distant from other huge phages based on MCP phylogeny and protein family analyses (Figures S5 and S6).

In Lak phages with complete genomes, different phylogenetic groups correlate with different protein family contents (Figure 5). Animal-specific protein families seem independent of geographic origins. The lack of functional predictions for these animal-specific proteins is interesting and points to adaptation to either the microbiome conditions or the specific host bacterium following dispersal of these phages among animal hosts. Moreover, non-detection of 660-kb Lak in animals other than horses suggests possible specificity to equine microbiomes. It is plausible that the large genomes of Lak phages may impact host range and extracellular viability (outside of host). Effects due directly to animal physiology are possible, but similarities in diet, and thus *Prevotella* species or strain composition of gut microbiomes most likely influences the distribution of Lak phages.

### Distribution of Lak phage and *Prevotella* across pig gastrointestinal sites

Pigs were used in this study to model the distribution of *Prevotella* and Lak phages throughout the monogastric digestive tracts. It should be noted that 1) Lak host range within the *Prevotella* genus is uncertain, so the broad *Prevotella* qPCR primers that were used (Zozaya-Hinchliffe et al., 2010) may not exclusively target the *Prevotella* spp. that Lak phages infect and 2) The exact 16s rRNA copy number per *Prevotella* genome in pig samples could not be determined (due to fragmented sequences for these genomes). Nonetheless, results provide an overview for the distribution of *Prevotella* and Lak phages, which aligns with the current knowledge that *Prevotella* is common in pigs, and are enriched in the hindgut (main site of fiber fermentation) compared to the foregut (*Firmicutes-* and *Proteobacteria*-dominated) (Isaacson and Kim, 2012; Liu et al., 2012; Looft et al., 2014; Ramayo-Caldas et al., 2016; Yang et al., 2016). Within the foregut and hindgut compartments, the absolute abundances of Lak and *Prevotella* genes in the lumen and mucosa do not differ at GIT sites (*P* < 0.05; Figure 1B), even though *Prevotella* can degrade mucins and are equipped to colonize the mucosa (Rho et al., 2005). Theoretically, the relative abundance of Lak phages compared to *Prevotella* might also be higher in the mucosa compared to the lumen because the adhesion of phages to the mucosa should increase phage-bacteria encounter rates (Barr et al., 2015; Lourenço et al., 2020). The finding that this is not the case may relate to the counteracting effect of the mucosa allowing bacteria to evade phage predation (Barr et al., 2015; Lourenço et al., 2020).

The ratio of Lak: *Prevotella* abundance was higher in the foregut compared to the hindgut mucosa (Figure 1C). This may be a consequence of slower digesta transit times through the hindgut compared to foregut lumens (Wilfart et al., 2007), coinciding with increased establishment and thus higher relative abundance of *Prevotella* in the foregut mucosa compared to other bacteria (Isaacson and Kim, 2012; Liu et al., 2012; Looft et al., 2014; Ramayo-Caldas et al., 2016; Yang et al., 2016). This may increase the probability of successful Lak phage replication in the foregut compared to the hindgut. Another consideration is that phage: host ratios can decrease as bacteria within a population acquire resistance or exhibit abortive infection, whereby infected host bacterium sacrifices itself, limiting phage infection to the remaining population (Lopatina et al., 2020). This was suggested to be the case in a study of *Staphylococcus epidermidis* and phages in the human infant gut (Sharon et al., 2013). It is also plausible that *Prevotella* strain variation between foregut mucosal tissues compared to other GIT sites influenced Lak: *Prevotella* ratios. It was not possible to find capsid protein domains linked to mucosal adaptation using the present annotation pipelines, although this has been reported in phages previously (Barr et al., 2013).

### Factors affecting the prevalence of Lak in pigs

Phenotypic differences in pig breeds, sex, and age can affect microbiome composition (Xiao et al., 2016, 2018). Lak-positive pigs represented a variety of breeds, ages, and both sexes. However, Lak was most frequently detected in finisher pigs (*n =* 13) and not detected in piglets or gestating sows. Colonization of the piglet GIT is facilitated by the sow through birth and lactation (Wang et al., 2013), but their microbiomes are highly unstable and a *Bacteroides* to *Prevotella* shift often occurs as maturity is reached (Mach et al., 2015; Pajarillo et al., 2014). Lak prevalence in finisher pigs could relate to the dietary provision of fibrous ingredients being greater than in other production stages but lower than in gestating sows (e.g. 17.5% wheat feed +5% rapemeal (Cambridge dry sow) vs. 5% wheat feed +7% rapemeal (Cambridge finisher); Table S7). This may have increased microbial diversity and reduced the proportion of *Bacteroidetes* in gestating sows compared to finisher pigs (Jiang et al., 2019; Mou et al., 2020). Overall, dietary differences that reduced *Prevotella* relative abundances may explain the non-detection of Lak in piglets and gestating sows (Table S7).

### Possible significance of *Prevotella* lysis

In accordance with previous findings (Devoto et al., 2019), the newly constructed Lak phage genomes do not possess identifiable integrases or show evidence of prophage in gut metagenomes. This points toward a virulent lifestyle. However, based on our qPCR analyses, we also demonstrate that Lak phages co-occur with *Prevotella* at pig gastrointestinal sites and relatively few virions exist compared to possible hosts. Similarly, genomic evidence for lysogeny is commonly absent in the extensively studied CrAssphage genomes, which may persist as dormant within host cells (Liang and Bushman, 2021). Given their large genome sizes and current evidence, it is unlikely that Lak phages integrate. Infection characteristics of huge phages are poorly understood and require further investigation.

Lak phage predation could shape *Prevotella* population structure and overall microbiome composition. This is important because, although a commensal in various microbiomes, *Prevotella* has been linked to a variety of human diseases (Gharbia et al., 1994; Gilbert et al., 2019; Maeda et al., 1998; Nagaoka et al., 2014; Pybus and Onderdonk, 1997; Ulrich et al., 2010; Zozaya-Hinchliffe et al., 2010). *P. copri* overgrowth in the gut has been linked to rheumatoid arthritis in humans (Alpizar-Rodriguez et al., 2019; Pianta et al., 2017; Scher et al., 2013). *P.* *bivia* is strongly associated with bacterial vaginosis (Gilbert et al., 2019; Pybus and Onderdonk, 1997; Zozaya-Hinchliffe et al., 2010), and recently severe pre-eclampsia in humans (Lin et al., 2020). We detected Lak phage in three pig vaginal mucosas, albeit at lower abundance than in rectums (Table S3). A similar Lak: *Prevotella* ratio between the vagina and rectum suggests comparable Lak replication. *P. copri* and *P. bivia* are common in both pigs (Amat et al., 2020) and humans (Alauzet et al., 2010; Amat et al., 2020), thus it is possible that Lak predation of these bacteria could reduce the incidence of their associated diseases. Further study of Lak host range is required but represents challenges given the difficulty of culturing huge phages.

In humans and animals, *Prevotella* lysis by Lak phages may affect fiber fermentation, with potential health implications. In pigs, *Prevotella*-dominated enterotypes are associated with improved growth performance (Mach et al., 2015; Ramayo-Caldas et al., 2016). Given that *Prevotella* are enriched in the hindgut where fiber is primarily fermented, lysis could be detrimental to the animal host. However, overgrowth of certain *Prevotella* species in pigs may reduce feed efficiency and facilitate undesirable fat accumulation (Quan et al., 2020; Tan et al., 2017; Chen et al., 2020a). Thus, Lak phage predation could positively or negatively impact swine production. Besides the presence of a cecum, the pig gut physiology and microbiome composition are comparable to humans (Xiao et al., 2016). Therefore, the distribution of Lak phage and *Prevotella* in the swine GIT could inform our understanding of Lak and *Prevotella* distributions more generally.

### Conclusions

Lak phages are substantially more widespread and have a larger range of genome sizes and genome GC contents than previously realized. All lineages appear to use the same alternative genetic code. Lak phages occur in the microbiomes of many humans and animals including reptiles, with the largest detected in a racehorse. Conserved protein families suggest genomes adapted to specific animal microbiomes. Lak phages appear to be particularly common in pig microbiomes, where they are found in multiple body sites and enriched in the hindgut. It may be possible to harness Lak phages to modulate microbiome structure and composition, with long-term implications for the treatment of human diseases, including rheumatoid arthritis and vaginosis, and to improve swine growth performance.

### Limitations of the study

As detailed in this paper, multiple 16s rRNA copies in *Prevotella* genomes and uncertainty surrounding potential Lak host strains should be considered during interpretation of our qPCR data. Quantification of 660 kbp Lak and *Prevotella* in horse B over 3 days provides an overview, however, a longer sample period should be used in future investigations of Lak and *Prevotella* abundance over time. Furthermore, a large proportion of phage proteins currently have no functional annotations. This is exacerbated in larger phage genomes.

## STAR★methods

### Key resources table

**Table:** 

REAGENT or RESOURCE	SOURCE	IDENTIFIER
**Biological samples**		

See Table S2 for details of microbiome samples	This study	NA

**Chemicals, peptides, and recombinant proteins**		

*Prevotella copri* DNA	DSMZ	DSM 18205

**Critical commercial assays**		

QIAquick PCR purification kit	Qiagen	28,106
QIAquick gel extraction kit	Qiagen	28,706
QIAamp PowerFecal DNA kit	Qiagen	51,804
QuantiNova SYBR green PCR kit	Qiagen	208054
BIOTAQ™ DNA Polymerase	Bioline	BIO-21060

**Deposited data**		

Lak genomes	This paper	https://www.ncbi.nlm.nih.gov/bioproject/PRJNA688310
Lak genomes	This paper	https://ggkbase.berkeley.edu/Lak2/organisms
Lak genomes	This paper	https://ggkbase.berkeley.edu/Lak2/organisms

**Oligonucleotides**		

See Table S10 for Lak qPCR and PCR primers	This paper	
See Table S10 for *Prevotella* 16s rRNA qPCR primers	Zozaya-Hinchliffe et al. (2010)	

**Software and algorithms**		

JMP® Pro 14.1	SAS Institute Inc., NC, USA, 2019	https://www.jmp.com/en_gb/home.html
MEGA X	Kumar et al. (2018)	https://www.megasoftware.net
BLASTN 2.10.0+	Altschul et al. (1990)	https://ftp.ncbi.nlm.nih.gov/blast/executables/
Primer-BLAST	Ye et al. (2012)	https://blast.ncbi.nlm.nih.gov/Blast.cgi
Geneious Prime	Geneious	https://www.geneious.com/
Bowtie2 V2.3.5.1	Langmead and Salzberg (2012)	https://github.com/BenLangmead/bowtie2
Prodigal v2.6.3	Hyatt et al. (2010)	https://github.com/hyattpd/Prodigal
MEGAHIT v1.2.9	Li et al. (2015)	https://github.com/voutcn/megahit
IDBA_UD	Peng et al. (2012)	https://i.cs.hku.hk/∼alse/hkubrg/projects/idba_ud/
tRNAscan-SE	Chan and Lowe (2019)	https://github.com/UCSC-LoweLab/tRNAscan-SE
MMseqs2	Steinegger and Söding (2017)	https://github.com/soedinglab/MMseqs2
IQ-TREE 2	Minh et al., 2020	https://github.com/iqtree/iqtree2
HHpred	Remmert et al. (2011)	https://github.com/soedinglab/hh-suite
OligoAnalyzer	Integrated DNA Technologies Inc., Iowa, USA	https://www.idtdna.com/calc/analyzer

**Other**		

Sanger sequencing	LightRUN	Eurofins, Germany
Published Lak genomes	Devoto et al. (2019)	https://www.ncbi.nlm.nih.gov/bioproject/?term=PRJNA491720

All primers were synthesized by Sigma-Aldrich (MO, USA).

### Resource availability

#### Lead contact

Further information and requests for resources and reagents should be directed to and will be fulfilled by the lead contact, Prof. Joanne Santini (j.santini@ucl.ac.uk).

#### Materials availability

This study did not generate new unique regents.

#### Data and code availability

The 34 newly reconstructed Lak megaphage genomes have been deposited at NCBI under BioProject PRJNA688310, and also available from ggkbase https://ggkbase.berkeley.edu/Lak2/organisms (please sign in by providing your email address to download) and at figshare (https://figshare.com/articles/dataset/34_new_Lak_phage_genomes/13493721). The NCBI accession information for all published datasets is available from Table S1. Raw qPCR data is available in Table S8, and statistical outputs are reported in Table S9.

This paper does not report original code.

Any additional information required to reanalyze the data reported in this paper is available from the lead contact upon request.

### Experimental model and subject details

#### Animals and sampling

A total of 194 samples from different animals were screened by PCR. Available animal details, including ages, are reported in Table S2. In addition to fecal samples, digesta and mucosal tissues were obtained where possible. No animals were euthanized for the purpose of this study, and gut sampling followed approved institutional standard operating procedures. At Langford Abattoir (University of Bristol, UK), finisher pigs (*Sus scrofa*) (20–24 weeks) were fasted for 24-hr prior to arrival, where they were stunned and humanely slaughtered before gut, vaginal and lung sampling. Pigs 1–4 and 7–11 (Welsh x Petrain) came from a different smallholding to pigs 5–6 (Welsh). For GIT sampling, pigs were reared in pairs with 1 male and 1 female (1–2, 3–4, 5–6), and each pair was reared separately. Vaginal, lung and rectal samples from female finisher pigs 7–11 (20–24 weeks) were harvested separately. Pig feces from a commercial farm was obtained and supplied by The University of Cambridge, UK (Large white x Landrace x Hampshire). Cambridge samples pertained to various production stages: 2 piglets, 2 pre-farrow sows, 3 early-gestation sows, 1 late-gestation sow, 5 weaner pigs (8–12 weeks), 2 grower pigs (12–18 weeks), 2 finisher pigs (18–22 weeks).

Rumen-cannulated dairy cows (*Bos taurus,* Holstein) were also sampled (Center for Dairy Research, CEDAR, University of Reading, UK). Frozen ROSS 308 broiler (*Gallus gallus domesticus*) caecal digesta was obtained from a feeding trial at The Royal Veterinary College (RVC, UK). Only samples from untreated, control birds were used. Available animal diet composition is listed in Table S7.

### Method details

#### Sample collection

To avoid cross-contamination, gloves were changed between each sample and only sterile equipment and collection tubes were used. For all fecal samples, approximately 2 g of feces was taken from the center of the sample to limit environmental contaminants. Dairy cows at CEDAR were moved to individual pens and cannulas opened for rumen fluid collection. Rectal samples were also taken from the same 3 cows. Cambridge pig samples were collected in sterile 7 mL tubes and frozen at −80°C, before transfer to The Santini Lab, University College London (UCL, UK) on dry ice. All other feces, cow and broiler digesta were transferred on ice packs to UCL within 3 hr and stored at −80°C until analysis. Most fecal samples were collected at a single time point, but Epsom racehorse samples (Horse A-F; Wendover stables, UK) were collected for 3 consecutive days.

At Langford abattoir, (after scalding) entire GITs from 6 post-mortem finisher pigs (1–6) were removed from esophagus to rectum, within 30 min of slaughter. Digestive compartments were sectioned with cable ties and removed: mid-jejunum, terminal ileum (10 cm anterior to ileo-caecal junction), proximal spiral (10 cm distal to ileo-cecal junction), distal spiral, distal cecum and rectum. Luminal digesta and mucosal scrapings were collected using ethanol-sterilized equipment. Ileal lumens were empty in 2/6 pigs. Vaginal and rectal samples from pigs 7–11 were also obtained before scalding, vulvas were sanitized with 100% ethanol and vaginal mucosas were removed using sterile equipment, rectal samples were then collected using clean spatulas. Lung sampling from the same animals was carried out post-scalding; tracheas were clamped to avoid scalding lung contents before longitudinal dissection of each lung following the left and right bronchi. Mucosal scrapings of each lung were taken with sterile scalpels and pooled for each pig. All post-mortem samples were flash-frozen and transported on dry ice to UCL and stored at −80°C.

#### DNA extraction

All samples were thawed at room temperature and DNA was extracted using a QIAamp PowerFecal DNA kit (Qiagen, Hilden, Germany), following the manufacturer instructions. DNA concentration and 260/280 Ratio were measured in duplicate using NanoDrop™ 2000 (ThermoFisher Scientific, MA, USA) and averaged, to ensure sufficient DNA quality and concentration for PCR.

#### PCR and amplicon sequencing

The genes for major capsid protein (MCP), portal vertex protein (PVP) and tail sheath monomer (TSM) from human and baboon Lak genomes were aligned using ClustalW in MEGA-X (Kumar et al., 2018) to identify homologous regions. Primers were designed in Primer-BLAST (Ye et al., 2012) and synthesized by Sigma-Aldrich (MO, USA). The designed primer pairs were specificity checked and optimized (Table S10).

Each 25 μL PCR reaction contained 150 ng/μL template DNA (alongside a swine positive control), 5.5 μL master mix, free deoxynucleotides (dNTPs, 200 μM), forward and reverse primers (0.14 μM), NH_4_ reaction buffer (1 ×), and MgCl_2_ (3 mM), and 1.25 U BIOTAQ™ DNA Polymerase (Bioline, London, UK). A Mastercycler Nexus GSX1 (Eppendorf, Germany) was programmed for 40 cycles with DNA denaturation temperature of 96°C for 10 s, annealing (MCP: 61°C, PVP: 58°C, TSM: 57°C, 660kbp_MCP: 57°C) for 30 s, and extension at 72°C (MCP and PVP: 15s, TSM: 20s, 660kbp_MCP: 25s) with a final extension of 10 min. PCR amplicons were visualized by agarose gel electrophoresis. PCR products were purified using either a QIAquick PCR purification or gel extraction kit (Qiagen, Hilden, Germany). Sanger sequencing of purified PCR products was performed by Eurofins, Germany. BLASTN (Altschul et al., 1990) was used to confirm sequences were similar to Lak. Forward and reverse sequences were aligned using MEGA-X (Kumar et al., 2018), and quality checked against sequence chromatograms. In general screening, three genes (Lak MCP, TSM and PVP) were sequenced for all animal cohorts, except giant tortoise (GTA), fallow deer (FD), pig 2 jejunal mucosa (JM2) and horse B (HB2) where two of the three PCR products were sequenced. For the 660 kb Lak, only MCP sequences were obtained for all four horses. A summary of sequences obtained for each sample is reported in Table S1.

#### Quantitative real-time PCR (qPCR)

Lak phage and *Prevotella* abundances were determined by quantitative PCR (qPCR) using the standard curve method. *Prevotella* genus-specific 16s rRNA primers designed previously for the human vaginal microbiome were used (Zozaya-Hinchliffe et al., 2010). Primer-BLAST (Ye et al., 2012) was used to check coverage for common *Prevotella* species. These included strains of *P. copri*, *P. stercorea*, *P. melaninogenica*, *P. intermedia*, *P. jejuni*, *P. bivia* and *P. nigrescens*, many of which are found in pigs and humans (Alauzet et al., 2010; Amat et al., 2020). For pig GIT experiments, Lak MCP genes from available pig metagenomes (Devoto et al., 2019) were aligned by ClustalW in MEGA-X (Kumar et al., 2018). The MCP gene from the 660 kbp genome identified in Horse B alone was also used to design primers. Lak candidate primers with amplicons 114–221 bp were designed in Primer-BLAST (Ye et al., 2012) and synthesized by Sigma-Aldrich (MO, USA), along with *Prevotella* primers. Primer pairs were checked for primer dimers and hairpins in OligoAnalyzer (Integrated DNA Technologies Inc., Iowa, USA) and specificity-checked by Sanger sequencing PCR products prior to use in qPCR (Eurofins, Germany).

For pig GIT qPCR, LakMC581-F/LakMC1053-R PCR product from pig rectal DNA was used to generate Lak standards, as this encompassed qPCR targets. For horse 660 kbp Lak qPCR, LakHMC185-F/LakHMC984-R product from horse B fecal DNA was used to generate Lak standards. *P. copri* DNA (DSM, 18205, type strain) was used for *Prevotella* standards. Serial dilutions (9 × 1:10) starting at 5 ng DNA were used for standard curves (quantification cycle (Cq) vs. Log DNA dilution) during quantification and to determine primer efficiencies ((-1 + 10^−1/slope^) x 100). The selected qPCR primer pairs are reported in Table S10. For pig GIT qPCR, the selected Lak primer pair yielded an efficiency of 102.8%. For the horse 660 kbp Lak qPCR, the selected Lak primer pair yielded an efficiency of 110.1%. The *Prevotella* primer pair (Zozaya-Hinchliffe et al., 2010) (used in both experiments) yielded an efficiency of 94.1%.

qPCR was performed using a PikoReal™ real-time PCR system (Thermo Fisher Scientific, MA, USA), with a QuantiNova SYBR Green PCR kit (Qiagen, Germany). 9 uL master mix providing 1× SYBR Green master mix, 0.7 uM primers, 1× ROX passive reference dye and 1 uL nuclease-free water, was pipetted into Piko 96-well plates (Thermo Fisher Scientific, MA, USA). 1 uL gDNA (providing 10 ng in each reaction), diluted in 1 × template dilution buffer, was added to the master mix (10 uL reaction volume). Plates were sealed using Piko Optical Heat Seals (Thermo Fisher Scientific, MA, USA). Standards were run in parallel to sample DNA, and three technical replicates and no template controls (NTC; water in place of DNA) were included throughout. Primer efficiencies remained at an acceptable range of 90–110%, and melt curves suggested no non-specific binding or secondary structures (Figure S7). Lak and *Prevotella* quantities (ng) were extrapolated from standard curves and collated.

#### Metagenomic sequencing and analyses

A total of 31 samples confirmed with Lak phages were sequenced. The raw reads of each metagenomic sample were filtered to remove Illumina adapters, PhiX and other contaminants with BBTools (Bushnell, 2018), and low-quality bases and reads using Sickle (version 1.33, https.github.com/najoshi/sickle). The high-quality reads of each sample were assembled using idba_ud (Peng et al., 2012) (parameters: --mink 20 --maxk 140 --step 20 --pre_correction), or MEGAHIT (Li et al., 2015) (parameters: --k-list 21,29,39,59,79,99,119,141). For a given sample, the high-quality reads of all samples from the same sampling site were individually mapped to the assembled scaffold set of each sample using Bowtie 2 with default parameters (Langmead and Salzberg, 2012). The coverage of a given scaffold was calculated as the total number of bases mapped to it divided by its length. The scaffolds with a minimum length of 1 kbp were uploaded to the ggKbase platform. The protein-coding genes were predicted using Prodigal (Hyatt et al., 2010) (-m -p meta) from scaffolds and annotated using usearch (Edgar, 2010) against KEGG (Kanehisa et al., 2017), UniRef (Suzek et al., 2007) and UniProt (Apweiler et al., 2004). Some published metagenomic datasets (Table S1) (Munk et al., 2018; Pasolli et al., 2019) were also analyzed using the same pipeline as described above.

#### Manual genome curation

The *de novo* assembled contigs/scaffolds were searched against the 15 published Lak genomes (Devoto et al., 2019) using BLASTN (Altschul et al., 1990). To get Lak contigs/scaffolds candidates for genome curation, the BLAST hits were filtered to retain those with an alignment longer than 2 kbp and a minimum similarity of 90%. Notably, the resulting curated genomes may have a much lower genome-wide similarity with previously published genomes. The target contigs/scaffolds from a given sample were grouped into bin(s) based on their GC content and coverage. Manual genome curation was performed on the bin(s) as previously described (Chen et al., 2020b) by read mapping, scaffold extension and join, and manual fixation of assembly errors, attempt for completion was also conducted until a circularized genome was obtained. The determination of a complete genome was generally based on “circular” signal via reads mapping, no ‘core’ gene set was used for evaluation. We also validated that the automatic virus sequence identification tool VIBRANT (Kieft et al., 2020) could identify LAK contigs, and other tools including virFinder (Ren et al., 2017) and VirSorter (Roux et al., 2015) were also able to detect LAK contigs as viruses, as we could find Lak fragments in the dataset reported recently (Camarillo-Guerrero et al., 2021).

#### CRISPR-Cas analyses

All the predicted proteins of scaffolds with a minimum length of 1 kbp were searched against local HMM databases including all reported Cas proteins, and the nucleotide sequences of the same set of scaffolds were scanned for CRISPR loci using minced (Bland et al., 2007) (-minSL = 17). The spacers were extracted from the scaffolds with CRISPR loci as determined by minced, and also from reads mapped to these corresponding scaffolds using a local python script as previously described (Chen et al., 2019). For the published genomes, only spacers from the scaffold consensus sequences were extracted, as no mapped reads are available. Duplicated spacers were removed using cd-hit-est (-c = 1, -aS = 1, -aL = 1) and the unique spacer sequences were used to build a database for BLASTN (Altschul et al., 1990) searches (task = blastn-short, e-value = 1 × 10^−3^) against the Lak genomic sequences. Once a spacer was found to target a Lak phage scaffold with at least 90% alignment similarity, the original scaffold of the spacer was checked for a CRISPR locus and Cas proteins.

#### tRNA analysis

The tRNAs were predicted using tRNAscan-SE (Chan and Lowe, 2019) in eukaryotic mode with default settings. Lak tRNAs have been previously established to contain introns and thus are not all classified in bacterial mode.

#### Phage protein family analyses

All the 34 new reconstructed Lak phage genomes, and the 15 published Lak genomes from human and baboon gut microbiomes (Devoto et al., 2019), and two recently published genomes (Edgar et al., 2020) from Dog gut microbiomes (Allaway et al., 2020) were included for protein family analyses, which were performed as previously described (Méheust et al., 2019). In detail, first, all-vs-all searches were performed using MMseqs2 (Steinegger and Söding, 2017), with parameters set as e-value = 0.001, sensitivity = 7.5 and cover = 0.5. Second, a sequence similarity network was built based on the pairwise similarities, then the greedy set cover algorithm from MMseqs2 was performed to define protein subclusters (i.e., protein subfamilies). Third, in order to test for distant homology, we grouped subfamilies into protein families using an HMM-HMM comparison procedure as follows. The proteins of each subfamily with at least two protein members were aligned using the result2msa parameter of MMseqs2, and HMM profiles were built from the multiple sequence alignment using the HHpred suite (Söding et al., 2005). The subfamilies were then compared to each other using hhblits (Remmert et al., 2011) from the HHpred suite (with parameters -v 0 -p 50 -z 4 -Z 32000 -B 0 -b 0). For subfamilies with probability scores of ≥95% and coverage ≥0.5, a similarity score (probability × coverage) was used as the weights of the input network in the final clustering using the Markov CLustering algorithm (Enright et al., 2002), with 2.0 as the inflation parameter. Finally, the resulting clusters were defined as protein families. The clustering analyses of the presence and absence of protein families detected in the phage genomes were performed with Jaccard distance and complete linkage.

#### Phylogenetic analyses

To reveal the phylogeny of Lak phages reconstructed in this study. The shared single-copy gene product sequences from each genome were concatenated and aligned with MAFFT (default parameters) (Katoh et al., 2019). The alignment was subsequently converted into a phylogenetic tree on the MAFFT web-server using 100 bootstraps, Neighbor Joining, JTT as a substitution model. For other single gene phylogenetic analyses, the corresponding protein sequences were aligned using MUSCLE (Edgar, 2004) with default parameters, the alignment was then filtered using trimAl (Capella-Gutiérrez et al., 2009) to remove those columns with >90% gaps. The phylogenetic trees were built by IQtree (Minh et al., 2020) using the ‘GTR + G4’ model with 1000 bootstraps.

### Quantification and statistical analysis

#### qPCR data analysis

Copy numbers were calculated, log-transformed, and technical replicates averaged (Table S8). The qPCR data were analyzed in JMP Pro 14.1 (SAS Institute Inc., NC, USA, 2019) (Table S9). For the pig GIT qPCR, distribution was analyzed by ‘GIT site’ for both mucosal and lumen log copy numbers. No outliers were identified 1.5∗IQR. A Shapiro Wilk-Test for normality suggested data were near normally distributed (*P* > 0.05). To compare Lak phage abundances, standard least square mean comparisons were made using a full factorial approach and restricted maximum likelihood (REML) method, across the 6 biological replicates. ‘GIT site∗Sample type’, ‘Sex’ and ‘Farm’ were included as fixed effects, and plate number as a random effect, to account for co-variation. Actual vs. predicted values indicated adequate model fit (R^2^ = 0.96, RMSE = 0.36, *P* < 0.0001). The abundance of Lak phage copies: *Prevotella* copies were calculated to estimate phage copies per host 16s rRNA gene, a fixed effect model was used with the same parameters, but plate number was omitted. Treatment means were separated using Tukey's HSD test (α = 0.05 and 0.001). Least square comparisons were made between vagina and rectal samples with no co-variates (as these animals were of the same sex, from the same farm, and qPCR was performed on a single plate), and a Student's *t* test was used. For the horse Lak qPCR, copy numbers for Lak 660 kbp Lak MCP and *Prevotella* 16s rRNA genes from horse B were plotted, and the correlation coefficient determined using a 95% density ellipse. As the 660 kbp Lak was only found in one of the Epsom racehorses (horse B, Wendover stables), there were no replicates. Therefore, qPCR results from horse G (single day, different stables) were plotted in parallel to validate results from horse B.

## Supplemental information

Document S1. Figures S1–S6

Table S1. General information of samples with PCR checking, PCR product sequencing, metagenomic sequences and genome reconstruction, related to Tables 1 and 2

Table S2. General details of the animals that were investigated in this study by PCR, related to Table 1

Table S3 Lak phage and Prevotella abundance differs in the pig vagina and rectum, related to Table 1 and Figure 1 (1)Copy number calculated from absolute quantification of Lak major capsid protein and Prevotella 16s rRNA genes using qPCR, per 10 ng microbiome gDNA. Anti-log means are presented, but log copy numbers were analyzed, ranging from 4.01–6.00 with standard errors of *Prevotella* = 0.15 and Lak Phage = 0.09. Data represents 3 female finisher pigs. Lak was undetectable from the lungs of the same animals. See statistical outputs in Table S9.

Table S4. 660 kbp Lak phage shows day-to-day variation, related to Table 1 (1)Lak phage and Prevotella copy numbers determined by absolute quantification qPCR, with 10 ng pooled DNA from Horse B feces collected over 3 consecutive days. Lak primers targeted the major capsid gene of 660 kbp Lak genome, and Prevotella primers targeted the 16S rRNA gene. A sample from Horse G at a single time point was run in parallel to validate results; and copy numbers were comparable to those observed in Horse B. r = correlation coefficient indicates that there was not a strong correlation between Lak phage and Prevotella abundance over the 3 sample days. Anti-log means are presented, but log copy numbers were analyzed, ranging from 2.98–6.67. See statistical outputs in Table S9.

Table S5. The tRNAs identified in the scaffolds of each high-quality Lak genome, related to Figure 4 The supressor tRNAs are highlighted (TTA supressor tRNA in blue, and CTA supressor tRNA in orange).

Table S6. The presence and absence and the annotation of protein families identified for Lak megaphages and other huge phages, related to Figure 5

Table S7. Available dietary information for animal diets investigated in this study, related to Table 1

Table S8 The raw data of qPCR results for pig and horse samples investigated in this study, related to Figure 1 and Table 1

Table S9 The JMP model outputs for all qPCR data, related to Figure 1 and Table 1

Table S10 PCR and qPCR oligonucleotide primers used in the present study, related to Figure 1 and Table 1
