# Supplementary material for: Closely related Lak megaphages replicate in the microbiomes of diverse animals
Source: iScience. 2021 Jul 16;24(8):102875. doi: 10.1016/j.isci.2021.102875 (PMC8346664; doi:10.1016/j.isci.2021.102875)
Supplement: Document S1. Figures S1–S6 [file mmc1.pdf]

iScience, Volume *24*

Supplemental information

Closely related Lak megaphages replicate in the microbiomes of diverse animals

Marco A. Crisci, Lin-Xing Chen, Audra E. Devoto, Adair L. Borges, Nicola Bordin, Rohan Sachdeva, Adrian Tett, Allison M. Sharrar, Nicola Segata, Francesco Debenedetti, Mick Bailey, Rachel Burt, Rhiannon M. Wood, Lewis J. Rowden, Paula M. Corsini, Steven van Winden, Mark A. Holmes, Shufei Lei, Jillian F. Banfield, and Joanne M. Santini

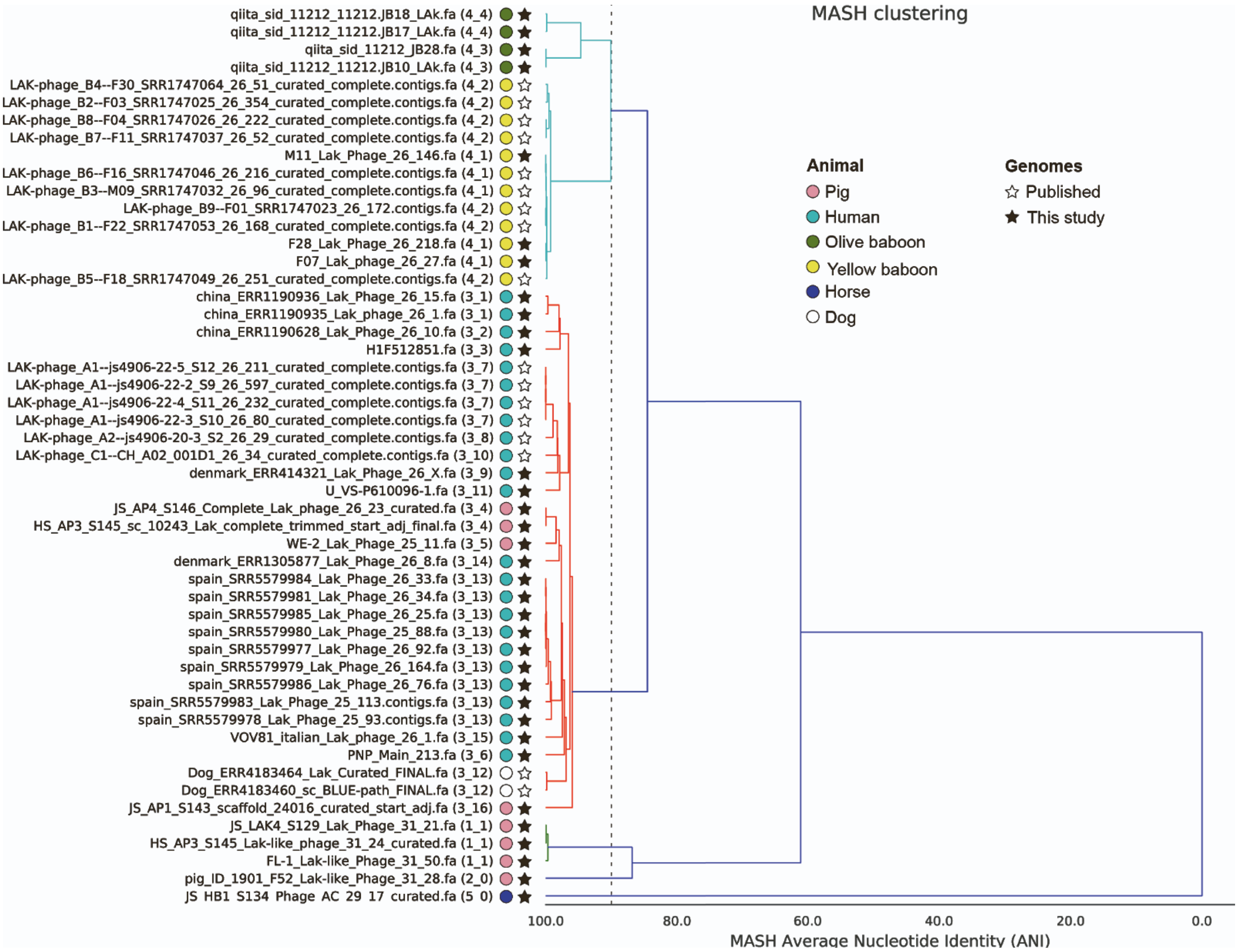

Figure S1 (Related to Table 2) | The genome wide similarity of new reconstructed and published Lak phages.

The results were generated by dRep with the input parameters as following: -sa 0.99 -nc 0.5 --ignoreGenomeQuality. Note that dRep could not give a similarity value for the one from Horse against others as it is too divergent.

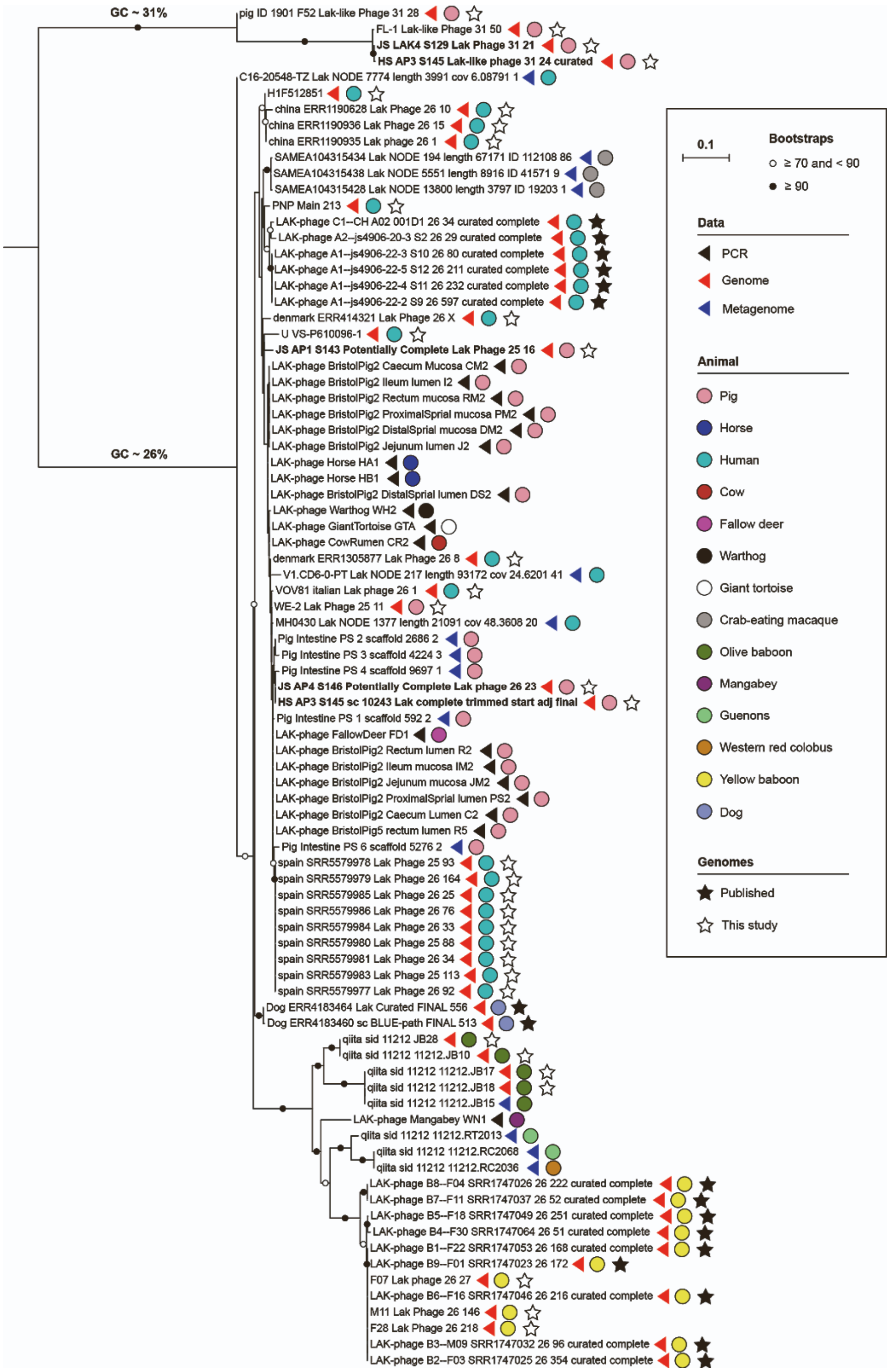

Figure S2 (Related to Figure 2) | Lak phages from diverse animals are phylogenetically related. Phylogeny was based on sequences from PCR, genomes and metagenomes. The nucleotide sequences encoding the portal vertex protein (PVP) were aligned, and trimmed based on the length of the PCR sequences. The PVP of the ~660 kbp phage is very divergent from others, thus was excluded from the tree to enable resolution of the other sequences. The tree was rooted between the GC31 group and GC26 group, according to the full phylogeny of all Lak (including the ~660 kbp one) and some other published huge phages (Figure S6). The names of the complete Lak genomes reported in this study are in bold. Bristol pig sequences obtained from the vaginal mucosa were identical to those found in the digestive tract.

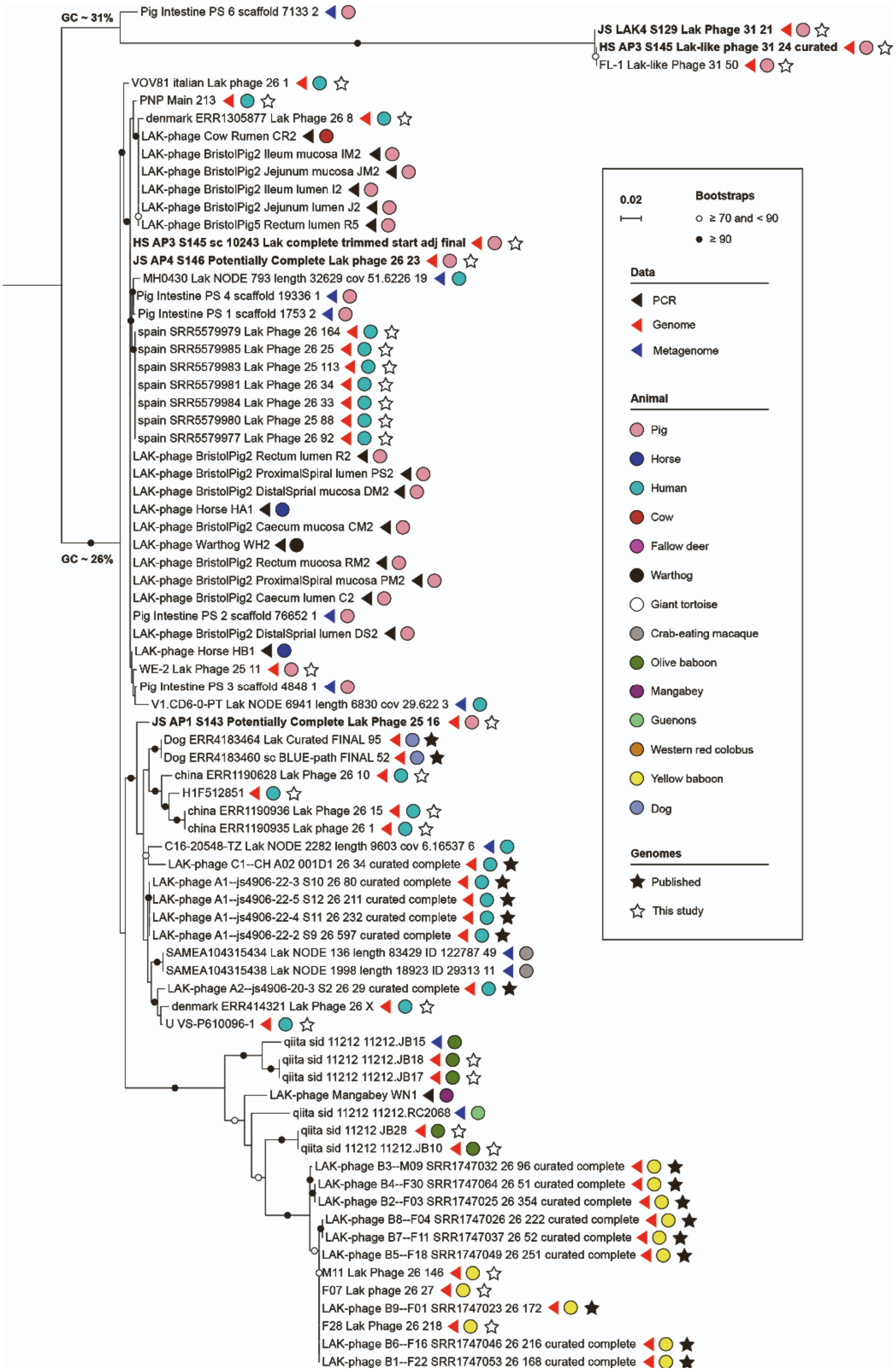

Figure S3 (Related to Figure 2) | Lak phages from diverse animals are phylogenetically related. Phylogeny was based on sequences from PCR, genomes and metagenomes. The nucleotide sequences encoding the tail sheath monomer (TSM) were aligned, and trimmed based on the length of the PCR sequences. The TSM of the ~660 kbp phage is very divergent from others, thus was excluded from the tree to enable resolution of the other sequences. The tree was rooted between the GC31 group and GC26 group, according to the full phylogeny of all Lak (including the ~660 kbp one) and some other published huge phages (Figure S6). The names of the complete Lak genomes reported in this study are in bold. Bristol pig sequences obtained from the vaginal mucosa were identical to those found in the digestive tract.

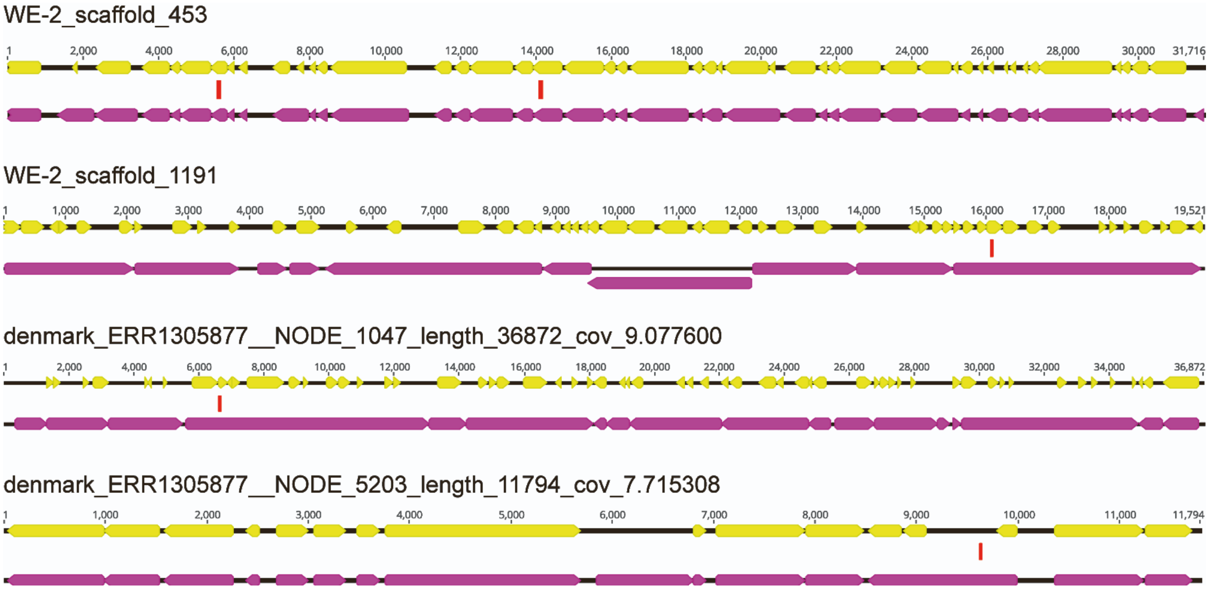

Figure S4 (Related to Table 2) | Newly reported Lak phages predicted to replicate in *Prevotella*. Examples of Lak phage sequences targeted by CRISPR-Cas spacers from *Prevotella* spp. The targeted fragments by spacers are shown by red bars. For each scaffold, the protein-coding genes predicted with code 11 are shown in yellow, and those predicted with code 15 are shown in pink.

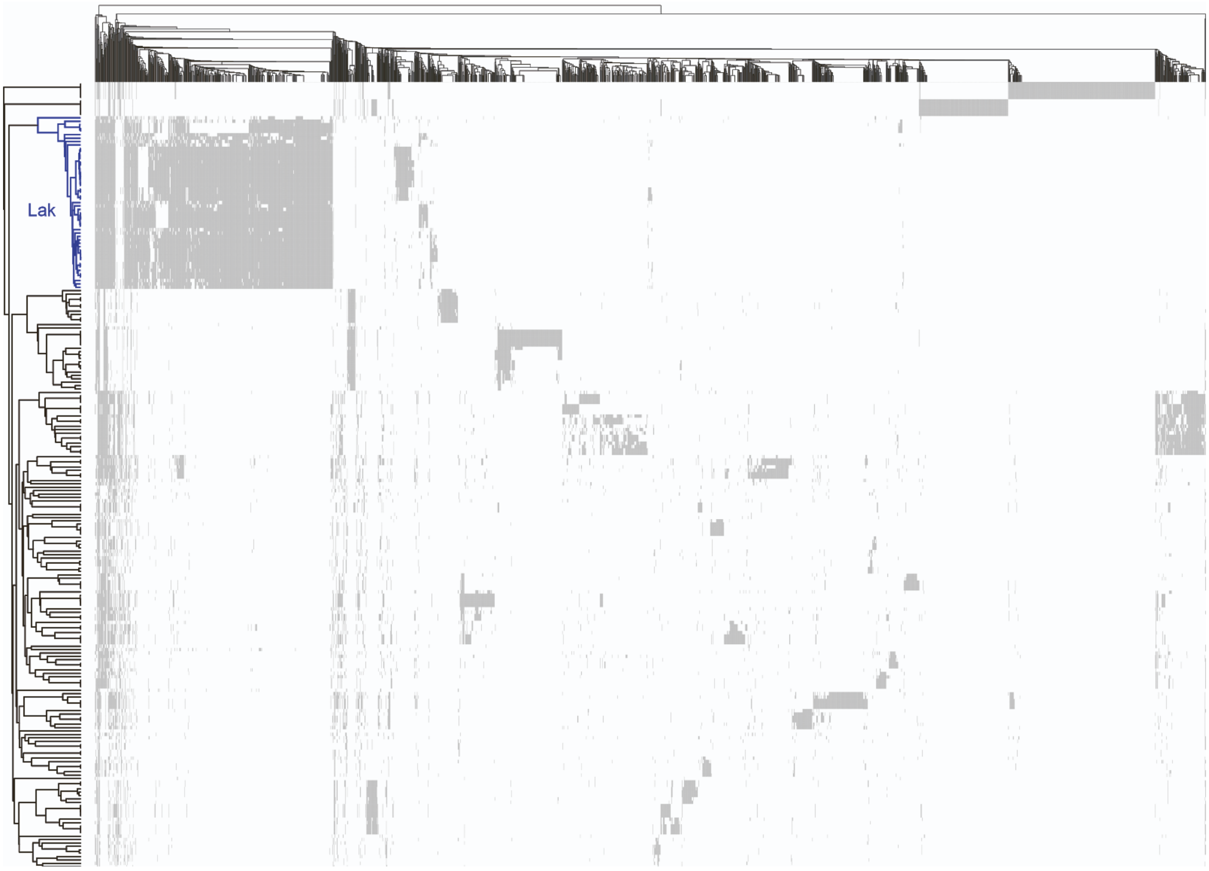

Figure S5 (Related to Figure 5 and Table 2) | The clustering analyses of 51 Lak phages (including 17 published ones and 34 new ones reported in this study) and 181 huge phages reported with circular genomes based on the presence and absence of protein families.

Only those protein families detected in four or more Lak phage genomes were included. The clustering analyses were performed with Jaccard's distance and complete linkage.

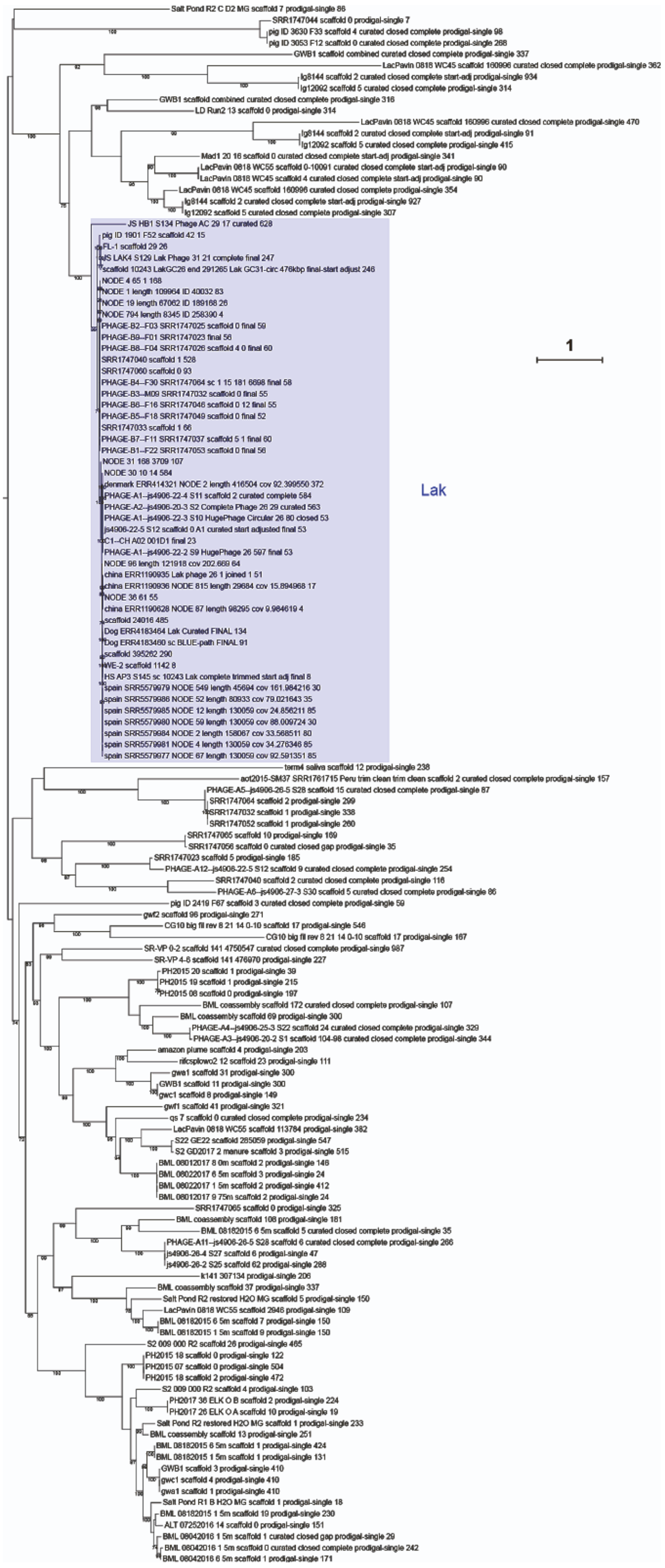

Figure S6 (Related to Figure 5 and Table 2) | The phylogeny of Lak phages and published huge phages.

The phylogeny was built based on the major capsid proteins (MCP) revealed by protein family analyses, the published huge phage genomes (retrieved from Al-Shayeb et al., 2020).

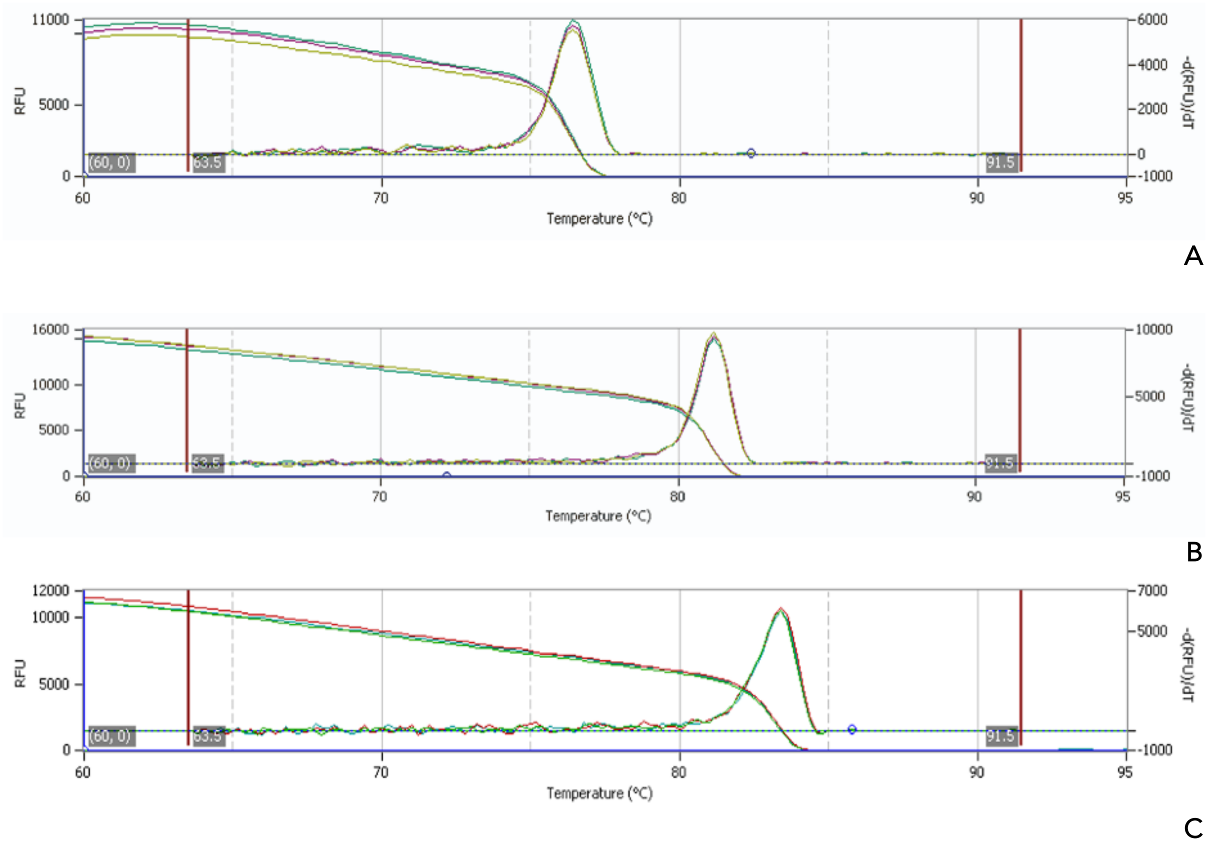

Figure S7 (Related to Table 1 and Figure 1) | Representative melt curves for selected qPCR primer pairs.

RFU=relative fluorescence units using SYBR green. Single peak generated from 3 technical replicates indicates no nonspecific binding or secondary structures. (**A**) Representative result shown for Lak major capsid gene primers designed for pig GIT experiment, using 10 ng Pig 2 Proximal Spiral (PS2) pooled digesta DNA. (**B**) Representative result shown for Lak major capsid gene primers designed for Horse 660 kbp variant experiment, using 10 ng Horse B (HB2) pooled faecal DNA. (**C**) Representative result shown for *Prevotella* genus-specific 16s rRNA primers designed previously (Zozaya-Hinchliffe et al., 2010) using 10 ng Pig 2 Rectal (R2) pooled digesta DNA. See qPCR primer details in Table S10.
